# Cryptococcosis and Cryptococcal Meningitis: A Narrative Review and the Up-to-Date Management Approach

**DOI:** 10.7759/cureus.55498

**Published:** 2024-03-04

**Authors:** Zaheer A Qureshi, Haider Ghazanfar, Faryal Altaf, Ali Ghazanfar, Khushbu Z Hasan, Sameer Kandhi, Ked Fortuzi, Arundhati Dileep, Shitij Shrivastava

**Affiliations:** 1 Medicine, Frank H. Netter MD School of Medicine, Quinnipiac University, Bridgeport, USA; 2 Internal Medicine, BronxCare Health System, New York City, USA; 3 Internal Medicine, Federal Medical and Dental College, Islamabad, PAK; 4 Internal Medicine, Mohtarma Benazir Bhutto Shaheed Medical College, Mirpur, PAK; 5 Gastroenterology and Hepatology, BronxCare Health System, New York City, USA; 6 Critical Care Medicine, Montefiore Medical Center, Bronx, USA; 7 Medicine, California Institute of Behavioral Neurosciences & Psychology, Fairfield, USA

**Keywords:** preventive and social medicine, fluconazole, amphotericin, encephalitis, meningitis, immunosuppression, hiv, cryptococcosis, antiretroviral therapy, antifungal therapy

## Abstract

Cryptococcosis is a fungal infectious disease that enormously impacts human health worldwide. Cryptococcal meningitis is the most severe disease caused by the fungus Cryptococcus, and can lead to death, if left untreated. Many patients develop resistance and progress to death even after treatment. It requires a prolonged treatment course in people with AIDS. This narrative review provides an evidence-based summary of the current treatment modalities and future trial options, including newer ones, namely, 18B7, T-2307, VT-1598, AR12, manogepix, and miltefosine. This review also evaluated the management and empiric treatment of cryptococcus meningitis. The disease can easily evade diagnosis with subacute presentation. Despite the severity of the disease, treatment options for cryptococcosis remain limited, and more research is needed.

## Introduction and background

**Figure 1:**
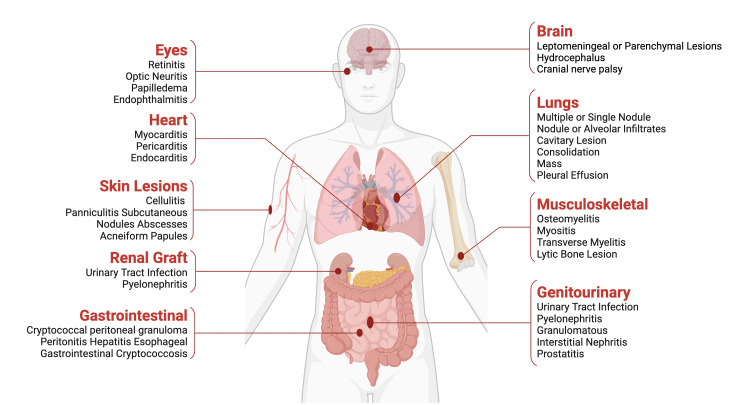
Involvement of various organs in a cryptococcal infection Image created using Biorender.com.

Cryptococcosis is an invasive opportunistic fungal infection caused by *Cryptococcus neoformans *or *Cryptococcus gattii* that has become increasingly prevalent in immune-compromised patients, and is either acquired or hereditary. *C. neoformans* is the principal pathogenic member of the genus and has a worldwide distribution. There are around one million cases of cryptococcosis globally each year, with 625,000 deaths reported [1,2]. The incidence of cryptococcal meningitis (CM) was 223,100 cases globally in 2014 [1,2]. It is challenging to accurately assess the incidence of cryptococcosis in the United States (US) as only a few states report the data. The case fatality ratio is reported to be approximately 12% [3-5]. Most cases are from Sub-Saharan Africa (162,500), followed by the Asia-Pacific region, the Caribbean, Latin America, North America, North Africa, the Middle East, and Europe. Three susceptible patient groups of cryptococcosis that are identified include HIV-infected, transplant recipients, and HIV-negative/non-transplant patients [6]. Non-HIV-related/non-transplant-related risk factors include glucocorticoid therapy, neoplasia, especially hematologic, liver disease and cirrhosis, sarcoidosis, and other autoimmune diseases [7]. Cryptococcosis commonly affects the central nervous system (CNS) and lungs, but the involvement of other systems has also been reported. Common CNS manifestations include headache, nausea, vomiting, general fatigue, encephalopathy, neck stiffness, photophobia, seizures, fever with associated signs, increased intracranial pressure (ICP), hearing loss, and papilledema (Figure 1). There has been a poor correlation between ICP and positive neuroimaging in patients with cryptococcal disease with CNS involvement [8]. This is why lumbar puncture (LP) and the opening pressure are paramount in suspected cases of CNS cryptococcosis, as they are significant risk factors for increased morbidity and mortality. Other organ systems like skin, eyes, and prostate are rarely affected, and there are few cases of orthopedic cryptococcosis, cryptococcal peritonitis, and gastrointestinal involvement [1,7,8].

The reported prevalence estimate of the cryptococcus disease among people with HIV was one million cases per year, with 625,000 deaths [9]. *Cryptococcus neoformans* is a significant cause of illness in people living with HIV/AIDS, with an estimated 0.22 million cases of cryptococcal meningitis occurring worldwide each year. T-cell-mediated immunity is required for anti-cryptococcal protection. The clearance of *Cryptococcal neoformans* is determined by the development of a robust CD4+ response TH1 > TH2. In many circumstances, the yeast will establish a latent infection within the macrophages, resulting in a latent infection within the thoracic lymph nodes or a pulmonary granuloma that can persist in asymptomatic individuals for years. As soon as the cellular immunity is compromised, the yeast can disseminate either as a yeast form or within the phagocyte to other body sites by direct invasion of the blood-brain barrier via transcytosis of free yeast forms through a series of mechanisms between yeast and host factors and transport via macrophages into the CNS (the “Trojan horse” mechanism) [10,11]. Before anti-retroviral therapy (ART), fungal and other opportunistic infections were a huge burden for the HIV/AIDS population. Since the advent of ART, the number has gone substantially down in the US and other developed countries with easy access to ART (one study showed a decrease of 90% in cases in the 1990s) [2,8,10].

## Review

Method

We searched online databases, including PubMed, Scopus, and Embase, for articles related to *Cryptococcus* and cryptococcus meningitis from inception till Feb 2024. We included clinical trials, meta-analyses, clinical trial extensions, subgroup analyses, post hoc analyses, cost-effectiveness analyses, and new human data.

Management of cryptococcal disease

**Figure 2:**
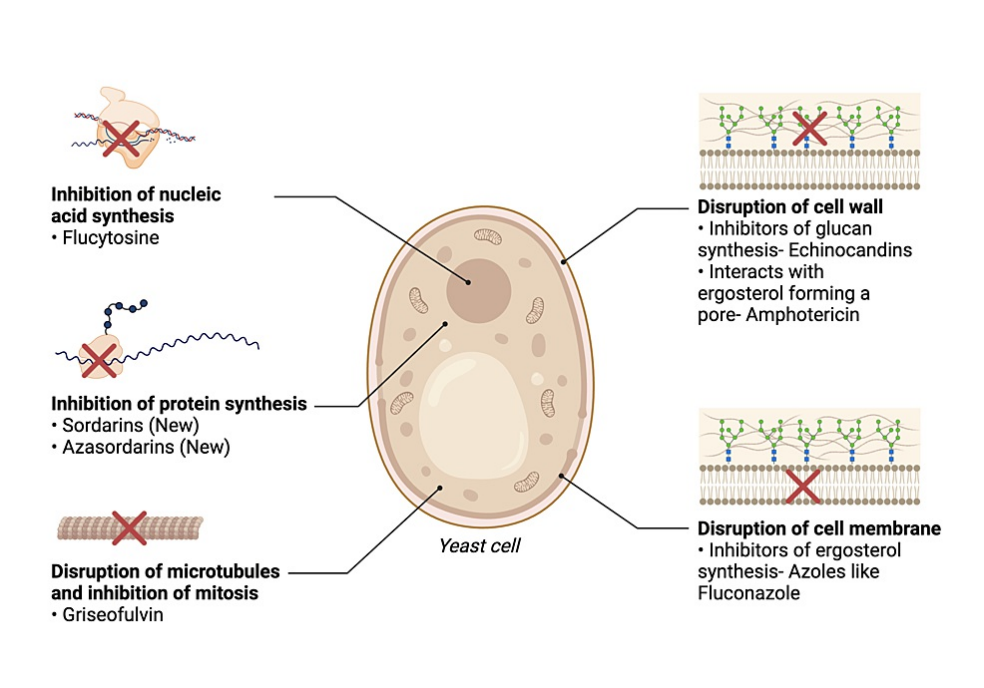
Mechanism of action of antifungal drugs Image created using Biorender.com.

Cryptococcosis is an infection that can affect both immunocompetent and immunocompromised hosts. The two main causative species of yeast that have been identified are *Cryptococcus neoformans *and *Cryptococcus gattii*. The most common human body sites affected by cryptococcal infection include the pulmonary and central nervous systems. Cryptococcal meningoencephalitis is one of the most common manifestations of CNS infection seen in patients with HIV [12]. Effective cryptococcal meningoencephalitis management typically involves prolonged antifungal treatment and ICP-lowering strategies. The therapy goals for cryptococcal disease include eradication of the infection and preventing dissemination of the infection. The antifungal treatment regimens include agents used individually or in combination for the treatment of cryptococcosis, and these include mainly amphotericin B (AmB), 5-flucytosine (5-FC), and fluconazole (azole group) [13]. Their mechanism of action is shown in Figure 2.

Amphotericin B

**Figure 3:**
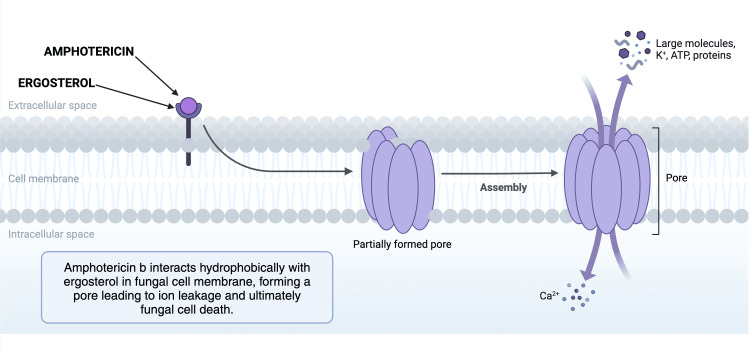
Mechanism of action of amphotericin B Image created using Biorender.com.

AmB is an antifungal agent that disrupts the fungal cell wall synthesis by binding to the sterols in the fungal cell membrane and forming pores that allow leakage of the cellular components, as shown in Figure 3. It is a fungicidal agent against many fungal organisms like *Candida *spp.,* Aspergillus *spp.,endemic mycoses, and *Cryptococcus *spp. [14]. The AmB molecule has an amino group and an amphoteric carboxyl group, whereas the conjugated heptaene on the macrolactone ring and the hydroxyl group make it amphiphilic. Due to its molecular structure, it is poorly soluble in water and even less soluble in salty solutions, making it difficult to administer it to patients [15].

5-Flucytosine

5-FC is an antifungal agent that inhibits protein and DNA synthesis. It is transported into the fungal cell by an enzyme known as cytosine permease. The metabolites of the medication include 5-fluoro-deoxy uridylic acid monophosphate, 5-fluorouridine monophosphate, and 5-fluorouridine diphosphate. These metabolites are crucial in inhibiting DNA and protein synthesis in the fungal cell. Depending on the fungal organism, 5-FC can act as static and cidal agents [16]. The absence of the abovementioned enzyme in mammalian cells makes it a selective antifungal agent.

Fluconazole

Fluconazole interferes with the fungal cytochrome P450 activity, reduces sterol synthesis, and inhibits cell membrane formation of the fungal cell by increasing permeability and causing cell lysis, and in turn, death [17].

Phases of treatment for cryptococcal meningitis

**Figure 4:**
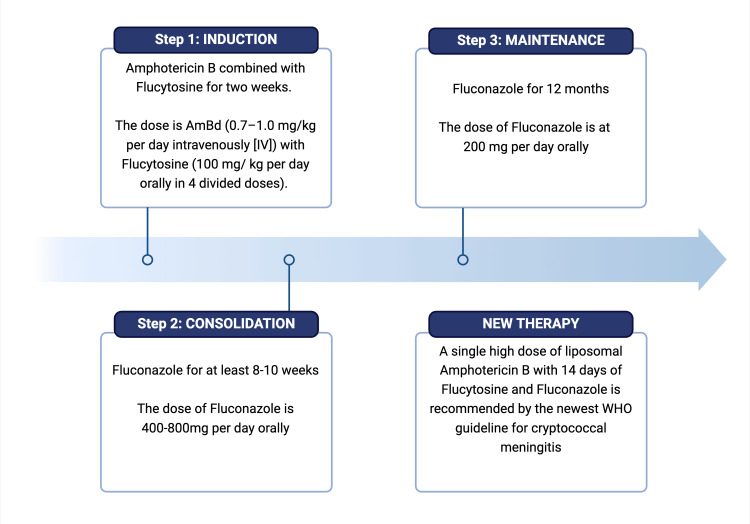
Latest treatment algorithm for cryptococcal meningitis Image created using Biorender.com.

The treatment for cryptococcal meningitis is divided into three phases: induction, consolidation, and maintenance therapy, as shown in Figure 4 [18].

Induction Therapy

The induction phase aims to drastically reduce the cerebrospinal fluid (CSF) fungal burden within the first two weeks. This is crucial for good patient outcomes and ensures lower chances of treatment failure [19]. For induction, first-line agents used in cryptococcal infections are amphotericin B combined with flucytosine for two weeks [19]. AmB) deoxycholate (AmBd) is the recommended first-line formulation. The dose for AmBd is 0.7-1.0 mg/kg per day intravenously (IV) with flucytosine (100 mg/ kg per day orally in four divided doses). IV formulations can be used for two weeks in patients with severe infections who cannot tolerate oral medications. Lipid formulations are preferred due to their lesser nephrotoxicity in comparison to other formulations of amphotericin B. Liposomal AmB can be used at 3-4 mg/day IV or AmB lipid complex (5 mg/kg per day IV for at least two weeks) and can be used instead of the regular AmBd in patients with renal dysfunction [20]. Recently, an alternative regimen has shown non-inferiority compared to the standard-of-care regimen where high-dose fluconazole (1200 mg/day) is combined with 5-FC for two weeks during the induction phase [21]. Many ongoing trials explore the concept of shorter duration for the induction phase and a combination of different molecules. At present, more data is needed to implement that in clinical practice. A similar treatment regimen is used for immunocompetent hosts with no HIV or solid transplant but who develop cryptococcosis; the difference is that the induction period is four weeks and can extend up to six weeks instead of the standard two weeks [19].

Consolidation

Once the patient receives induction therapy and an appropriate clinical response is obtained, we can transition them to consolidation therapy followed by maintenance therapy. The consolidation phase comprises orally giving fluconazole 400-800 mg (6 mg/kg per day) for at least 8-10 weeks [20].

Maintenance

After completing the consolidation, patients can be switched over to maintenance therapy with oral fluconazole at 200 mg/day for 12 months. A reduction in relapse rates of infections was found when patients received maintenance antifungal therapy during the first year of diagnosis. If the CD4 counts are maintained over 100 cells/μl, and the HIV viral load is undetectable for three months, maintenance therapy can be discontinued after 12 months [22]. Fluconazole was found to superior to other agents like itraconazole and AmB for maintenance [23]. In contrast, agents like voriconazole (VRCZ), posaconazole, and isavuconazonium, which have shown efficacy against *Cryptococcus*, are very costly alternatives and are used sparingly. They have demonstrated an improved activity against* Cryptococcus gattii *genotypes compared to fluconazole [24]. The consolidation and maintenance therapy in patients who are immunocompetent hosts with no HIV and no solid transplant but develop cryptococcosis is the same as for the ones who are immunocompromised. Initiating ART in these patients with CM is challenging as there is an increased risk of developing immune reconstitution syndrome. Many meta-analyses showed that ART when started within four weeks of diagnosis of cryptococcal meningitis, was associated with increased mortality [25].

Adjunctive Therapy

Patients with increased ICP, especially those with a CSF opening pressure of 25 cmH_2_O or more and cryptococcal meningitis, have shown improvement with therapeutic LP to reduce the opening pressure to ≤20 cmH_2_O [21].

Treatment of Localized Manifestations of Cryptococcal Disease

Other localized manifestations include pulmonary, cryptococcosis, and non-meningeal and non-pulmonary infections. In immunosuppressed and immunocompetent patients, mild to moderate pulmonary disease can be treated with fluconazole (400 mg/day) for 6-12 months [21]. Cryptococcomas can involve the lungs, brain, or both. The *Cryptococcus gattii* species are responsible for cryptococcosis, and they require a six-week induction phase with AmB and 5-FC followed by oral fluconazole (400-800 mg/day) for 6-18 months [21,22]. Large cerebral cryptococcomas might also need surgical excision [26]. On the contrary, cryptococcomas involving only the lungs can be treated with oral fluconazole 400 mg/day for 6-12 months [22]. Similarly significant, multiple pulmonary cryptococcomas require induction therapy with AmBd and 5-FC for a duration of four to six weeks, followed by consolidation and maintenance therapy with oral fluconazole for 6-18 months [22,26].

Novel therapies for *Cryptococcus*

Voriconazole

VRCZ, an extended-spectrum triazole, has been studied to treat cryptococcal infections. It acts by inhibiting 14-α lanosterol demethylation, thus preventing ergosterol synthesis, a major sterol in cell membranes of fungi. This action allows it to be fungistatic for most yeast species and fungicidal for some mold species [27]. *Cryptococcus*, a thick-walled yeast, demonstrates a spectrum of susceptibility to VRCZ. It was found that at subinhibitory concentrations in the Sabouraud dextrose medium (SAB), VRCZ did not affect the growth of *C. neoformans*, but it did alter capsular dimensions, as evidenced by electron microscopic imaging. When *C. neoformans* strain 24067 was incubated in the medium independently, the capsular volume was 635.5±225.7, while incubation with 0.5 minimum inhibitory concentration (MIC) of VRCZ resulted in a capsular volume of 137.8±59.4 (p<0.001) [28]. This has led to the theory that the inhibition of ergosterol in the cell membrane affects vesicular movement, which is needed for capsule synthesis [29].

Given amphotericin B's high efficacy against *C. neoformans*, voriconazole has not been adopted as a first-line treatment. However, the current literature supports the use of voriconazole in patients with AmB-resistant infections [28]. A case report by Serraj et al. demonstrated the efficacy of voriconazole monotherapy in systemic cryptococcus granulomatosis. The dose recommendations are 6 mg/kg IV every 12 h on Day 1, followed by a maintenance dose of 300 mg every 12 h orally [29,30]. Direct proportionality exists between increased voriconazole concentrations and elevated transaminases. This established 5.0 microgram/mL as the maximum serum voriconazole level [30].

Sertraline

Given challenges in drug development, drug repurposing has gained significant traction. Sertraline, a selective serotonin reuptake inhibitor, has been subject to such repurposing studies after it demonstrated potent in vitro fungicidal activity against *Cryptococcal neoformans*, synergistic to fluconazole [31]. The mechanism of anti-cryptococcal action has been purported to inhibit protein synthesis via the interaction with the eukaryotic translation initiation factor (Tif3) [32]. From March 2015 to May 2017, two hospitals in Uganda conducted a novel study that analyzed the potential use of sertraline as adjunctive therapy for cryptococcal meningitis in HIV patients. Modeled as a double-blinded, randomized clinical trial, this study compared the use of sertraline as adjunctive therapy versus placebo in patients being treated with IV amphotericin B and oral fluconazole, for cryptococcal meningitis. The primary endpoint was 18-week survival, and 460 patients were enrolled in this study: 229 patients in the sertraline group and 231 patients in the placebo group. However, at the end of 18 weeks, 120 of the 229 patients in the sertraline group (52%) and 106 of the 231 patients in the placebo group had died (hazard ratio 1.21, 95% CI 0.93-1.57, p=0.15), and this study was then terminated for futility. It was found that sertraline did not reduce mortality and was recommended not to be used to treat HIV-associated cryptococcal meningitis [33].

This study comes after a dose-finding survey conducted in August 2013 through 2014 to further quantify the dose of sertraline in the treatment of HIV-affected cryptococcal meningitis cases among 172 Ugandans. The doses of sertraline tried in this study were escalating doses of 100, 200, 300, and 400 mg/day as induction therapy for two weeks, followed by consolidation therapy with 200 mg/day through eight weeks. Based on pharmacokinetic projections, the brain concentration of sertraline would likely exceed MICs reported in vitro when dosed at 400 mg/day. At 400 mg/day, 81% of persons would achieve therapeutic sertraline activity in the brain. It was noted that there was no statistical difference in the early fungicidal activity (EFA) between doses of sertraline. Higher doses did not reduce the duration of hospitalization or mortality. However, the small size of this study resulted in overlapping confidence intervals [34].

Tamoxifen

Tamoxifen's mechanism of action when it comes to *Cryptococcus *is through the drug's binding to calmodulin and calmodulin-like proteins. This prevents the activation of calcineurin and thus prevents calcineurin-dependent nuclear localization, curbing the growth of *Cryptococcus* in macrophages [35]. In an open-label, randomized controlled trial, 50 patients with cryptococcal meningitis were studied to quantify the effect of tamoxifen on EFA (trial NCT03112031) [35,36]. Conducted in Vietnam, this study compared two groups of patients, those treated with standard therapy of AmB and fluconazole for the first two weeks and those treated with standard treatment plus 300 mg/day of tamoxifen. EFA was defined as the rate of yeast clearance from CSF. It was found that high-dose tamoxifen did not significantly affect the EFA [36].

Interferon Gamma

Adjunctive treatment with interferon gamma (IFNγ) was tried in a randomized clinical trial among patients with HIV-associated cryptococcal meningitis [37]. Patients were randomized into three groups: group 1 received standard therapy with AmB and 5-FC, group 2 received standard treatment plus IFNγ-1b (100 micrograms) on days 1 and 3, and group 3 received IFN gamma-1b (100 micrograms) on days 1, 3, 5, 8, 10 and 12. The primary outcome was the rate of cryptococcal clearance, measured by the EFA. Mean EFA (logCFU/ml/day) was −0.49 with standard treatment; −0.64 with IFNγ, two doses; and −0.64 with IFNγ, six doses. The difference in EFA was −0.15 (95% CI −0.02 to −0.27, p=0.02) between standard treatment and IFNγ (two doses), and −0.15 (95% CI −0.05 to −0.26, p=0.006) between standard treatment and IFNγ (six doses). As per the results of this study, there was a statistically significant increase in the clearance rate of cryptococcal viral particles from CSF with a short course of IFNγ therapy. The role of inherent IFNγ in the immune system's anticryptococcal processes has been demonstrated in multiple in vitro studies [38]. In these studies conducted on mice, IFN-gamma was instrumental in microglial cell activation and induction of CD40/IL2, which prevented the entry of cryptococcus in the cerebrospinal fluid.

In another randomized, double-blinded, placebo-controlled phase II trial conducted from January 2000 to July 2001, the safety and antifungal activity of recombinant IFNγ 1b (rIFNγ-1b) was further studied. In this study (trial NCT00012467), 60 patients with acute cryptococcal meningitis were divided into one of three parallel groups in a two-stage research over 14 weeks. In stage 1, patients were hospitalized for two weeks of acute therapy, wherein they received rIFNγ-1b or placebo subcutaneously three times per week, IV AmB daily, with or without oral 5-FC every six hours. In stage 2, patients received 84 days of consolidation therapy (56 days with study drug and 28 days of follow-up). The results of this study are yet to be published [39].

IFN-gamma cannot be used as monotherapy due to its toxic adverse effects, including flu-like illness, injection site reactions, and gastrointestinal symptoms [40]. Studies that sought to explore the pharmacotherapeutics of IFN-gamma also helped shed light on the possible therapeutic benefit of systemic treatment with interleukin 2 (IL-2) and anti-CD20 agents in preventing cerebral cryptococcal infection. This is mainly through the upregulation of major histocompatibility complex (MHC) class II in microglial cells, which then induces the phagocytic capacity of microglial cells [37-39]. The current literature contains a singular report of the successful treatment of cryptococcal meningitis with adjunctive recombinant IL-2 (rIL-2) [39,40]. The therapeutic role of the drug is yet to be studied in clinical trials.

Efungamab

NeuTec Pharma (a subsidiary of Novartis AG, Basel, Switzerland) developed an efungumab drug to treat candidemia in conjunction with IV AmB. This agent is a recombinant human antibody that binds to heat shock protein HSP90 on fungal particles. Fungal HSP90 is responsible for regulating thermotolerance in *Cryptococcus* and hence enables fungal growth. It is induced under the same conditions that cause capsule formation [41]. Biologic studies have revealed that interfering with HSP90 function improve anidulafungin (AF) tolerance in *Cryptococcus*; at 25°C and 37°C, an average growth of Cryptococcus was noted, indicating resistance to AF. When *Cryptococcus* was incubated with radicicol (an HSP90 inhibitor), no change in the fungal growth was reported at 25°C. Still, a 42% decrease in the growth was noted with a lower dose of AF (4 micrograms/mL). This supports the hypothesis that HSP90 is involved in thermotolerance and growth and that inhibitors of HSP90 can have additive effects with antifungal therapy [41,42].

Mycograb

In August 2006, Novartis sponsored a multicenter, randomized phase II clinical trial to study the efficacy and safety of Mycograb as adjunctive therapy for CM in patients with AIDS. Patients in this study were randomized into three groups of only standard treatment (with IV AmB and 5-FC), AmB with Mycograb, and standard therapy plus Mycograb. The results of this trial are yet to be published [43].

Morbidity and mortality in relation to cryptococcosis

Cryptococcosis is an invasive fungal disease that most commonly affects the CNS and lungs [44]. Still, also more rarely, other organ systems like skin, eyes, and prostate may be affected; there have been few reports of orthopedic cryptococcosis, cryptococcal peritonitis, and gastrointestinal involvement [45]. Pulmonary infection is associated with nonspecific findings like fever, chills, cough, malaise, night sweats, shortness of breath, weight loss, and rarely hemoptysis [46]. In immunocompetent hosts, the disease tends to remain confined in the lungs, but cases of disseminated disease have been documented [47,48].

Cryptococcal meningitis is a disease with significant morbidity and mortality that primarily affects the immunocompromised, but it can also affect immunocompetent hosts. Overall, the cases of CM have gone down since the advent of ART for AIDS. Still, there is an increased number of instances of solid-organ-transplant cases on immunosuppression, cancer chemotherapy, or people on long-term steroid use. Patients who survive CM may have long-term sequelae related to their infection, such as focal neurologic deficits, blindness, deafness, cranial nerve palsies, and memory deficits, and may require prolonged therapy or experience disease relapses [49]. Data regarding the real burden of the CM sequela is inconsistent, and not many studies have been done with that primary endpoint. As per a recent review, mortality in HIV-related CM can reach 78%, and in non-HIV CM 42%, after one year. Also, there is an essential long-term burden of CM on impairments and disability, with proportions reaching up to 70% in both HIV-infected and non-HIV-infected survivors [50].

Cryptococcus and HIV

CM is usually recognized by the innate immune system [51]. This system can effectively control the infection of a virulent *Cryptococcus *strain; however, the most pathogenic strains can rapidly upregulate factors that promote their growth. T-cell-mediated immunity is required for anti-cryptococcal protection [52]. The clearance of CM is determined by developing a robust CD4+ response TH1 > TH2. This is crucial for the development of an immune response. Still, there are highly virulent, rapidly disseminated strains that can result in meningitis and death, even in the presence of a robust TH1 response [53,54]. In many circumstances, the yeast will establish a latent infection within the macrophages, resulting in a latent infection within the thoracic lymph nodes or a pulmonary granuloma that can persist in asymptomatic individuals for years. As soon as the cellular immunity is compromised, the yeast can disseminate either as a yeast form or within the phagocyte to other body sites [55,56]. Both direct invasion of the blood-brain barrier via transcytosis of free yeast forms through a series of mechanisms between yeast and host factors and transport via macrophages into the CNS (the “Trojan horse” mechanism) seem to occur [56]. *C. neoformans* infections are rare among people who have healthy immune systems; however, *C. neoformans* is a significant cause of illness in people living with HIV/AIDS, with an estimated 220,000 cases of cryptococcal meningitis occurring worldwide each year [57]. Before ART, fungal and other opportunistic infections were a significant burden for the HIV/AIDS population, but with the advent of ART, the number has decreased substantially [58,59]. It is not easy to accurately estimate the incidence of cryptococcosis in the US, as only a few states report the data. Still, based on the surveillance done in 2000, the annual incidence of cryptococcosis was two to seven cases per 1000, with an overall incidence of 0.4-1.3 cases per 100,000 people [59]. The case fatality ratio was approximately 12% [59,60]. In cryptococcal meningoencephalitis, ART should be started at five weeks or later for patients with extremely low CD4 cell counts, CSF culture positive at two weeks despite the initiation of antifungal treatment, and those with a high fungal burden [60]. It is associated with improved survival compared to starting ART at one or two weeks [60].

Newer agents for the management of *Cryptococcus*

18B7

18B7 is a monoclonal antibody against a major *Cryptococcus neoformans* polysaccharide antigen component: glucuronoxylomannan (GXM). The monoclonal antibody causes deposition of the C3 complement, increases yeast cell phagocytosis, and has catalytic properties [61,62]. It can be administered as a single infusion with a maximum dose of 1 mg/kg. The monoclonal antibody has been shown to decrease serum cryptococcal antigen titers to half by a week and a third by two weeks. The adverse effects include myalgia, backache, and nausea at a 1 mg/kg dose [63].

T-2307

T-2307 is a structurally aromatic diamide like pentamidine. The exact mechanism by which T-2307 exerts its antifungal activity is unknown. The reduction of mitochondrial membrane potential might be an important factor [64]. It inhibits both fermentative and non-fermentative growth of *Cryptococcus neoformans* [65]. In murine systemic cryptococcosis, T-2307-treated mice had survival rates of 60%, 70%, and 100% at 0.1, 0.3, and 1 mg/kg, respectively, administered daily [66]. Although we could not find the results, a phase I trial of T-2307 has been completed (NCT02289599) [66,67].

VT-1598

VT-1598 is a tetrazole-based agent with high selectivity for fungal CYP51 enzyme and prevents ergosterol biosynthesis [66,67]. It shows broad-spectrum antifungal susceptibility, which includes *Candida*, *Cryptococcus*, and endemic fungal agents, with an in vitro study showing high susceptibility of *Cryptococcus neoformans* and *Cryptococcus gattii* [68]. In a murine model of cryptococcal meningitis, oral doses of VT-1598 showed a reduction in the fungal burden in six days [69]. A phase I trial of the drug is currently undergoing (NCT04208321).

AR12

AR12 is a celecoxib derivative that also has broad-spectrum antifungal activities. The antifungal properties are thought to derive from inhibiting the enzyme acetyl CoA synthase [69,70]. In a mouse model of cryptococcosis, the CFU per gram brain tissue after six days of treatment with AR12 and fluconazole was significantly less when compared with fluconazole alone, while AR12 alone had no significant effects [70]. A phase I trial of AR12 as an anticancer agent has been completed.

Manogepix

Manogepix is a fungal GWT1 inhibitor. Thus, the drug affects the maturation and localization of glycosylphosphatidylinositol (GPI)-anchored mannoproteins by inhibiting the inositol acetylation of fungal GPI proteins [69,70]. Manogepix has an in vitro and in vivo activity against *Cryptococcus*, and the effect is synergistic when combined with fluconazole [71]. Three phase I trials of manogepix have been completed so far; two did not report any clinically significant adverse effects or dose-limiting toxicities. A phase II trial has been completed for candidemia (NCT03604705). However, currently, to our knowledge, there are no trials for cryptococcosis [71,72].

Miltefosine

Miltefosine has antiprotozoal activities and has been widely used for leishmaniasis. Although the mechanism of the antifungal effects of miltefosine is not understood, its interaction with ergosterol may play a role [72]. The fungicidal effects of miltefosine against *Cryptococcus* were seen in an in vitro study with MIC values ranging from 0.5 to 2 microgram/mL [73].

## Conclusions

Cryptococcosis is a fungal infectious disease, and cryptococcal meningitis is its most serious form. CM is a rare clinical presentation but carries a 100% mortality rate if left untreated. There have been many trials on CM over the past 12 years, and the evidence base has never been more substantial. Many studies indicate the rising resistance to the traditional treatment method, especially azoles. Physicians must pay close attention to the diagnosis of CM, especially in immunocompromised patients, and early consultation with an infectious disease specialist is essential.

## References

[1] Rajasingham R, Smith RM, Park BJ (2017). Global burden of disease of HIV-associated cryptococcal meningitis: an updated analysis. Lancet Infect Dis.

[2] Casadevall A (2010). Cryptococci at the brain gate: break and enter or use a Trojan horse?. J Clin Invest.

[3] Mirza SA, Phelan M, Rimland D (2003). The changing epidemiology of cryptococcosis: an update from population-based active surveillance in 2 large metropolitan areas, 1992-2000. Clin Infect Dis.

[4] Kaplan JE, Hanson D, Dworkin MS (2000). Epidemiology of human immunodeficiency virus-associated opportunistic infections in the United States in the era of highly active antiretroviral therapy. Clin Infect Dis.

[5] Haddad NE, Powderly WG (2001). The changing face of mycoses in patients with HIV/AIDS. AIDS Read.

[6] McKenney J, Smith RM, Chiller TM, Detels R, French A, Margolick J, Klausner JD (2014). Prevalence and correlates of cryptococcal antigen positivity among AIDS patients--United States, 1986-2012. MMWR Morb Mortal Wkly Rep.

[7] Bratton EW, El Husseini N, Chastain CA (2012). Comparison and temporal trends of three groups with cryptococcosis: HIV-infected, solid organ transplant, and HIV-negative/non-transplant. PLoS One.

[8] Denning DW, Armstrong RW, Lewis BH, Stevens DA (1991). Elevated cerebrospinal fluid pressures in patients with cryptococcal meningitis and acquired immunodeficiency syndrome. Am J Med.

[9] Park BJ, Wannemuehler KA, Marston BJ, Govender N, Pappas PG, Chiller TM (2009). Estimation of the current global burden of cryptococcal meningitis among persons living with HIV/AIDS. AIDS.

[10] Charlier C, Nielsen K, Daou S, Brigitte M, Chretien F, Dromer F (2009). Evidence of a role for monocytes in dissemination and brain invasion by Cryptococcus neoformans. Infect Immun.

[11] Inoue H, Motohashi T, Ioku Y, Watanabe M, Nakajima M, Sugitatsu M (2020). The detection of Cryptococcus in skeletal infection after tooth extraction in an acute myeloid leukemia patient. IDCases.

[12] Perfect JR, Bicanic T (2015). Cryptococcosis diagnosis and treatment: what do we know now. Fungal Genet Biol.

[13] Olszewski MA, Zhang Y, Huffnagle GB (2010). Mechanisms of cryptococcal virulence and persistence. Future Microbiol.

[14] Hann IM, Prentice HG (2001). Lipid-based amphotericin B: a review of the last 10 years of use. Int J Antimicrob Agents.

[15] Falcón-González JM, Jiménez-Domínguez G, Ortega-Blake I, Carrillo-Tripp M (2017). Multi-phase solvation model for biological membranes: molecular action mechanism of amphotericin B. J Chem Theory Comput.

[16] Vermes A, Guchelaar HJ, Dankert J (2000). Flucytosine: a review of its pharmacology, clinical indications, pharmacokinetics, toxicity and drug interactions. J Antimicrob Chemother.

[17] Zonios DI, Bennett JE (2008). Update on azole antifungals. Semin Respir Crit Care Med.

[18] Kwon-Chung KJ, Fraser JA, Doering TL, Wang Z, Janbon G, Idnurm A, Bahn YS (2014). Cryptococcus neoformans and Cryptococcus gattii, the etiologic agents of cryptococcosis. Cold Spring Harb Perspect Med.

[19] Day JN, Chau TT, Wolbers M (2013). Combination antifungal therapy for cryptococcal meningitis. N Engl J Med.

[20] Perfect JR, Dismukes WE, Dromer F (2010). Clinical practice guidelines for the management of cryptococcal disease: 2010 update by the Infectious Diseases Society of America. Clin Infect Dis.

[21] Molloy SF, Kanyama C, Heyderman RS (2018). Antifungal combinations for treatment of cryptococcal meningitis in Africa. N Engl J Med.

[22] Bozzette SA, Larsen RA, Chiu J (1991). A placebo-controlled trial of maintenance therapy with fluconazole after treatment of cryptococcal meningitis in the acquired immunodeficiency syndrome. N Engl J Med.

[23] Saag MS, Cloud GA, Graybill JR (1999). A comparison of itraconazole versus fluconazole as maintenance therapy for AIDS-associated cryptococcal meningitis. Clin Infect Dis.

[24] Forrest GN, Bhalla P, DeBess EE, Winthrop KL, Lockhart SR, Mohammadi J, Cieslak PR (2015). Cryptococcus gattii infection in solid organ transplant recipients: description of Oregon outbreak cases. Transpl Infect Dis.

[25] Boulware DR, Meya DB, Bergemann TL (2010). Clinical features and serum biomarkers in HIV immune reconstitution inflammatory syndrome after cryptococcal meningitis: a prospective cohort study. PLoS Med.

[26] Franco-Paredes C, Womack T, Bohlmeyer T (2015). Management of Cryptococcus gattii meningoencephalitis. Lancet Infect Dis.

[27] Saravolatz LD, Johnson LB, Kauffman CA (2003). Voriconazole: a new triazole antifungal agent. Clin Infect Dis.

[28] van Duin D, Cleare W, Zaragoza O, Casadevall A, Nosanchuk JD (2004). Effects of voriconazole on Cryptococcus neoformans. Antimicrob Agents Chemother.

[29] Sakaguchi N, Baba T, Fukuzawa M, Ohno S (1993). Ultrastructural study of Cryptococcus neoformans by quick-freezing and deep-etching method. Mycopathologia.

[30] Serraj K Sr, Alaoui H, El Oumri AA, Barrimi M, Bachir H (2020). Effective voriconazole in an immunocompetent patient with amphotericin B resistant systemic cryptococcal granulomatosis. Cureus.

[31] Zhai B, Wu C, Wang L, Sachs MS, Lin X (2012). The antidepressant sertraline provides a promising therapeutic option for neurotropic cryptococcal infections. Antimicrob Agents Chemother.

[32] Rhein J, Huppler Hullsiek K, Tugume L (2019). Adjunctive sertraline for HIV-associated cryptococcal meningitis: a randomised, placebo-controlled, double-blind phase 3 trial. Lancet Infect Dis.

[33] Rhein J, Morawski BM, Hullsiek KH (2016). Efficacy of adjunctive sertraline for the treatment of HIV-associated cryptococcal meningitis: an open-label dose-ranging study. Lancet Infect Dis.

[34] Butts A, Koselny K, Chabrier-Roselló Y (2014). Estrogen receptor antagonists are anti-cryptococcal agents that directly bind EF hand proteins and synergize with fluconazole in vivo. mBio.

[35] Ngan NT, Thanh Hoang Le N, Vi Vi NN (2021). An open label randomized controlled trial of tamoxifen combined with amphotericin B and fluconazole for cryptococcal meningitis. eLife.

[36] Jarvis JN, Meintjes G, Rebe K (2012). Adjunctive interferon-γ immunotherapy for the treatment of HIV-associated cryptococcal meningitis: a randomized controlled trial. AIDS.

[37] Zhou Q, Gault RA, Kozel TR, Murphy WJ (2007). Protection from direct cerebral cryptococcus infection by interferon-gamma-dependent activation of microglial cells. J Immunol.

[38] Kullberg BJ (1997). Trends in immunotherapy of fungal infections. Eur J Clin Microbiol Infect Dis.

[39] Cheeran MC, Hu S, Sheng WS, Rashid A, Peterson PK, Lokensgard JR (2005). Differential responses of human brain cells to West Nile virus infection. J Neurovirol.

[40] Aravalli RN, Hu S, Rowen TN, Palmquist JM, Lokensgard JR (2005). Cutting edge: TLR2-mediated proinflammatory cytokine and chemokine production by microglial cells in response to herpes simplex virus. J Immunol.

[41] Suzuki Y, Claflin J, Wang X, Lengi A, Kikuchi T (2005). Microglia and macrophages as innate producers of interferon-gamma in the brain following infection with Toxoplasma gondii. Int J Parasitol.

[42] Yilmaz-Demirdag Y, Wilson B, Lowery-Nordberg M, Bocchini JA Jr, Bahna SL (2008). Interleukin-2 treatment for persistent cryptococcal meningitis in a child with idiopathic CD4(+) T lymphocytopenia. Allergy Asthma Proc.

[43] Chatterjee S, Tatu U (2017). Heat shock protein 90 localizes to the surface and augments virulence factors of Cryptococcus neoformans. PLoS Negl Trop Dis.

[44] Pasquier E, Kunda J, De Beaudrap P (2018). Long-term mortality and disability in cryptococcal meningitis: a systematic literature review. Clin Infect Dis.

[45] Pyrgos V, Seitz AE, Steiner CA, Prevots DR, Williamson PR (2013). Epidemiology of cryptococcal meningitis in the US: 1997-2009. PLoS One.

[46] Harris J, Lockhart S, Chiller T (2012). Cryptococcus gattii: where do we go from here?. Med Mycol.

[47] Nath DS, Kandaswamy R, Gruessner R, Sutherland DE, Dunn DL, Humar A (2005). Fungal infections in transplant recipients receiving alemtuzumab. Transplant Proc.

[48] Hage CA, Wood KL, Winer-Muram HT, Wilson SJ, Sarosi G, Knox KS (2003). Pulmonary cryptococcosis after initiation of anti-tumor necrosis factor-alpha therapy. Chest.

[49] Zavala S, Baddley JW (2020). Cryptococcosis. Semin Respir Crit Care Med.

[50] Chechani V, Kamholz SL (1990). Pulmonary manifestations of disseminated cryptococcosis in patients with AIDS. Chest.

[51] Rozenbaum R, Gonçalves AJ (1994). Clinical epidemiological study of 171 cases of cryptococcosis. Clin Infect Dis.

[52] Cameron ML, Bartlett JA, Gallis HA, Waskin HA (1991). Manifestations of pulmonary cryptococcosis in patients with acquired immunodeficiency syndrome. Rev Infect Dis.

[53] Meyohas MC, Roux P, Bollens D (1995). Pulmonary cryptococcosis: localized and disseminated infections in 27 patients with AIDS. Clin Infect Dis.

[54] Du L, Yang Y, Gu J, Chen J, Liao W, Zhu Y (2015). Systemic review of published reports on primary cutaneous cryptococcosis in immunocompetent patients. Mycopathologia.

[55] Hayashida MZ, Seque CA, Pasin VP, Enokihara MM, Porro AM (2017). Disseminated cryptococcosis with skin lesions: report of a case series. An Bras Dermatol.

[56] Boulware DR, Meya DB, Muzoora C (2014). Timing of antiretroviral therapy after diagnosis of cryptococcal meningitis. N Engl J Med.

[57] Casadevall A, Cleare W, Feldmesser M (1998). Characterization of a murine monoclonal antibody to Cryptococcus neoformans polysaccharide that is a candidate for human therapeutic studies. Antimicrob Agents Chemother.

[58] Bowen A, Wear MP, Cordero RJ, Oscarson S, Casadevall A (2017). A monoclonal antibody to Cryptococcus neoformans glucuronoxylomannan manifests hydrolytic activity for both peptides and polysaccharides. J Biol Chem.

[59] Larsen RA, Pappas PG, Perfect J (2005). Phase I evaluation of the safety and pharmacokinetics of murine-derived anticryptococcal antibody 18B7 in subjects with treated cryptococcal meningitis. Antimicrob Agents Chemother.

[60] Shibata T, Takahashi T, Yamada E (2012). T-2307 causes collapse of mitochondrial membrane potential in yeast. Antimicrob Agents Chemother.

[61] Wiederhold NP (2021). Review of T-2307, an investigational agent that causes collapse of fungal mitochondrial membrane potential. J Fungi (Basel).

[62] Mitsuyama J, Nomura N, Hashimoto K (2008). In vitro and in vivo antifungal activities of T-2307, a novel arylamidine. Antimicrob Agents Chemother.

[63] Hoekstra WJ, Garvey EP, Moore WR, Rafferty SW, Yates CM, Schotzinger RJ (2014). Design and optimization of highly-selective fungal CYP51 inhibitors. Bioorg Med Chem Lett.

[64] Hargrove TY, Garvey EP, Hoekstra WJ (2017). Crystal structure of the new investigational drug candidate VT-1598 in complex with Aspergillus fumigatus sterol 14α-demethylase provides insights into its broad-spectrum antifungal activity. Antimicrob Agents Chemother.

[65] Wiederhold NP, Lockhart SR, Najvar LK (2019). The fungal Cyp51-specific inhibitor VT-1598 demonstrates in vitro and in vivo activity against Candida auris. Antimicrob Agents Chemother.

[66] Garvey EP, Sharp AD, Warn PA, Yates CM, Schotzinger RJ (2018). The novel fungal CYP51 inhibitor VT-1598 is efficacious alone and in combination with liposomal amphotericin B in a murine model of cryptococcal meningitis. J Antimicrob Chemother.

[67] Koselny K, Green J, Favazzo L, Glazier VE, DiDone L, Ransford S, Krysan DJ (2024). Antitumor/antifungal celecoxib derivative AR-12 is a non-nucleoside inhibitor of the ANL-family adenylating enzyme acetyl CoA synthetase. ACS Infect Dis.

[68] Koselny K, Green J, DiDone L (2016). The celecoxib derivative AR-12 has broad-spectrum antifungal activity in vitro and improves the activity of fluconazole in a murine model of cryptococcosis. Antimicrob Agents Chemother.

[69] Watanabe NA, Miyazaki M, Horii T, Sagane K, Tsukahara K, Hata K (2012). E1210, a new broad-spectrum antifungal, suppresses Candida albicans hyphal growth through inhibition of glycosylphosphatidylinositol biosynthesis. Antimicrob Agents Chemother.

[70] Shaw KJ, Schell WA, Covel J (2018). In vitro and in vivo evaluation of APX001A/APX001 and other Gwt1 inhibitors against Cryptococcus. Antimicrob Agents Chemother.

[71] Hodges MR, Ople E, Wedel P (2023). Safety and pharmacokinetics of intravenous and oral fosmanogepix, a first-in-class antifungal agent, in healthy volunteers. Antimicrob Agents Chemother.

[72] Gushiken AC, Saharia KK, Baddley JW (2021). Cryptococcosis. Infect Dis Clin North Am.

[73] Spadari CC, Vila T, Rozental S, Ishida K (2018). Miltefosine has a postantifungal effect and induces apoptosis in Cryptococcus yeasts. Antimicrob Agents Chemother.

